# Supplementary material for: Proanthocyanidins Limit Adipose Accrual Induced by a Cafeteria Diet, Several Weeks after the End of the Treatment
Source: Genes (Basel). 2019 Aug 8;10(8):598. doi: 10.3390/genes10080598 (PMC6723337; doi:10.3390/genes10080598)
Supplement: Supplementary file 1 [file genes-10-00598-s001.pdf]

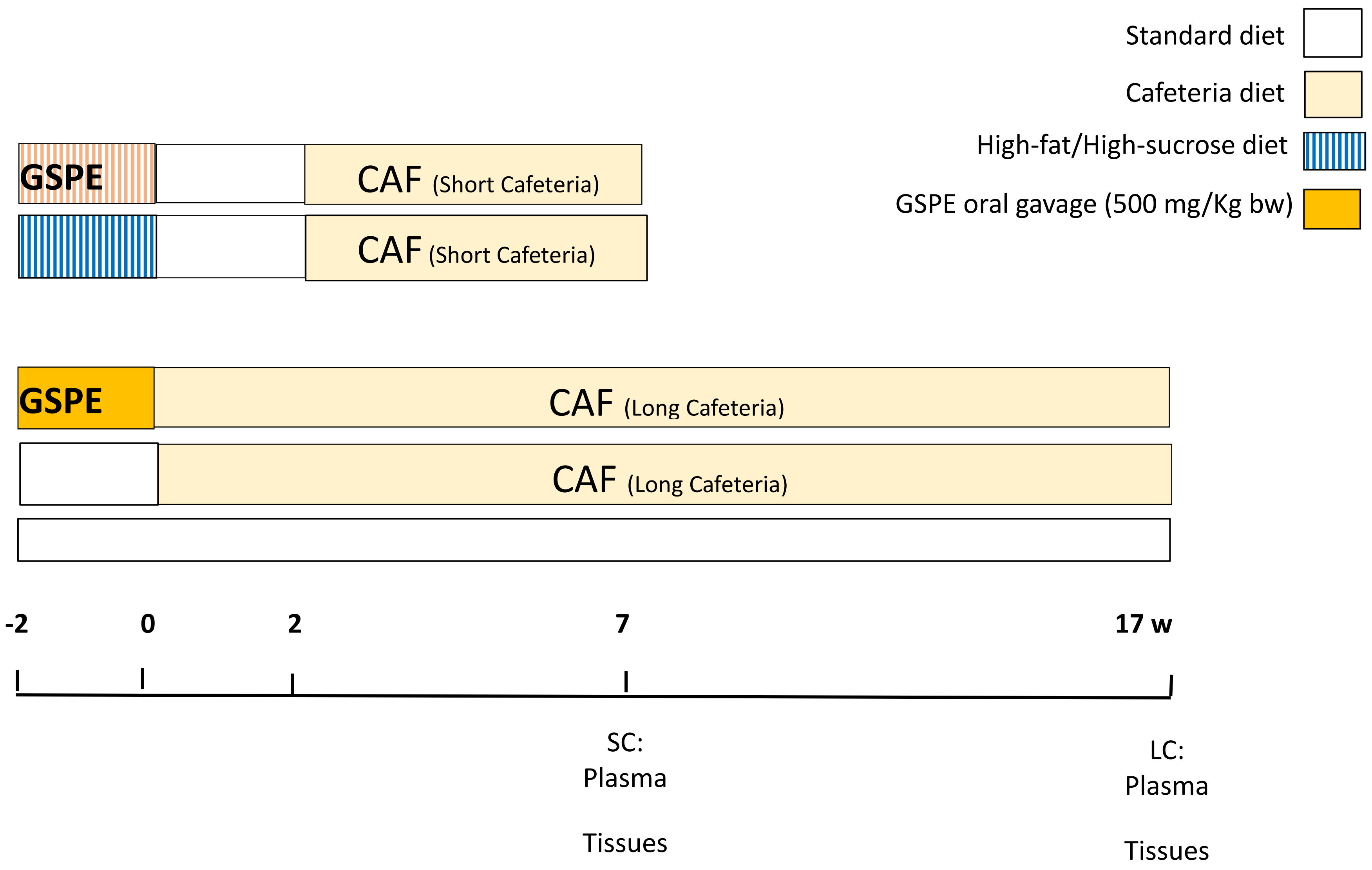
Standard diet
Cafeteria diet
High-fat/High-sucrose diet
GSPE oral gavage (500 mg/Kg bw)
GSPE
CAF (Short Cafeteria)
CAF (Short Cafeteria)
GSPE
CAF (Long Cafeteria)
CAF (Long Cafeteria)
-2
0
2
7
17 w
SC:
Plasma
Tissues
LC:
Plasma
Tissues

Supplementary Figure 1. **Schematic diagram of the experimental design**. (1) CAF-Short Cafeteria: rats receiving a GSPE preventive treatment for 10 days together with a high fat/high sucrose diet followed by an 18-day chow diet (standard diet) and then the 35-day cafeteria diet; (2) CAF-Long Cafeteria: rats receiving a GSPE preventive treatment 10 days before the 17-week cafeteria intervention. GSPE: grape seed proanthocyanidin extract; SC: Short Cafeteria; LC: Long Cafeteria.
